# Draft genome sequence of the type strain of the sulfur-oxidizing acidophile, *Acidithiobacillus albertensis* (DSM 14366)

**DOI:** 10.1186/s40793-017-0282-y

**Published:** 2017-12-15

**Authors:** Matías Castro, Ana Moya-Beltrán, Paulo C. Covarrubias, Mónica Gonzalez, Juan Pablo Cardenas, Francisco Issotta, Harold Nuñez, Lillian G. Acuña, Gonzalo Encina, David S. Holmes, D. Barrie Johnson, Raquel Quatrini

**Affiliations:** 1 Fundación Ciencia & Vida, Av. Zañartu 1482, Santiago, 7780272 Chile; 2 Genómica S.p.A, Santiago, Chile; 3 Facultad de Ciencias Biologicas, Universidad Andres Bello, Santiago, Chile; 4 College of Natural Sciences, Bangor University, Bangor, UK; 5 Current Address: uBiome Chile, SpA, Santiago, Chile

**Keywords:** Acidithiobacilli, Extreme acidophile, Sulfur oxidizer, Bioleaching, Sulfidic metal ores, Phylogenomics

## Abstract

*Acidithiobacillus albertensis* is an extremely acidophilic, mesophilic, obligatory autotrophic sulfur-oxidizer, with potential importance in the bioleaching of sulfidic metal ores, first described in the 1980s. Here we present the draft genome sequence of *Acidithiobacillus albertensis* DSM 14366^T^, thereby both filling a long-standing gap in the genomics of the acidithiobacilli, and providing further insight into the understanding of the biology of the non iron-oxidizing members of the *Acidithiobacillus* genus. The assembled genome is 3,1 Mb, and contains 47 tRNAs, tmRNA gene and 2 rRNA operons, along with 3149 protein-coding predicted genes. The Whole Genome Shotgun project was deposited in DDBJ/EMBL/GenBank under the accession MOAD00000000.

## Introduction

The genus 10.1601/nm.2198 [1] comprises a group of obligatory acidophilic chemolithotrophic bacteria that derive energy from the oxidation of reduced sulfur compounds, thereby contributing to the bioleaching of ores and to the formation of polluting mine drainage waters. Although they were considered until relatively recently as members of the Gamma-proteobacteria, multi-protein phylogenetic analysis of concatenated ribosomal proteins re-categorized the order 10.1601/nm.2196 as a new class of proteobacteria, now known as 10.1601/nm.24436 [2]. Currently, seven species are recognized: 10.1601/nm.2199 [3], 10.1601/nm.2202 [4], 10.1601/nm.2200 [5], 10.1601/nm.2201 [6], 10.1601/nm.17776 [7], 10.1601/nm.24751 [8], 10.1601/nm.27980 [9], four of which also catalyze the dissimilatory oxidation of ferrous iron while three (10.1601/nm.2199, 10.1601/nm.2200 and 10.1601/nm.2201 *)* do not.

Being capable of biogenic acid production and oxidation of reduced sulfur compounds, most species of the taxon have been exploited industrially in the recovery of valuable metals such as copper and gold and other relevant elements from ores and wastes ([10] and references therein). Not only are they frequent members of most analyzed bioleaching consortia, but tend also to be numerically relevant ([11] and references therein). Due to their biotechnological relevance most species of the taxon have been the object of intensive research since the early 1900’s [12]. Yet, despite compelling evidence regarding the widespread occurrence of 10.1601/nm.2200 [13–16] and its potential for chalcopyrite and sphalerite bioleaching [13, 17], 10.1601/nm.2200 remains the least studied species of all acidithiobacilli.

Whole genome sequences of a number of representative strains of four species of 10.1601/nm.2198 (10.1601/nm.2199 *,* 10.1601/nm.2202 *,* 10.1601/nm.2201 and 10.1601/nm.17776) have been reported to date [18] and genome comparisons have been performed both between and within species [19–23]. However, no representative genome sequence is yet available for 10.1601/nm.2200. Given that 10.1601/nm.2200 resembles 10.1601/nm.2199 in several aspects of their biology and physiology [5, 24], and that presence of either species in the natural and industrial environments tend to be confounded due to the high similarity between species at the 16S rRNA level [25], further characterization of the former is required to shed light into the species-specific processes. Availability of the whole-genome of the type strain of 10.1601/nm.2200 represents a first necessary step in this direction.

Here we present a description of the first draft of the genome sequence and annotation of the type strain of 10.1601/nm.2200 (10.1601/strainfinder?urlappend=%3Fid%3DDSM+14366 ^T^) along with relevant genomic indices of the taxon. The data presented fill a long-standing gap in the understanding of the genomic landscape of the acidithiobacilli and of the biology of 10.1601/nm.2200 and paves the way for more encompassing phylogenomic analyses of the species complex of these fascinating model acidophiles.

## Organism information

### Classification and features

Originally described by Bryant and colleagues [5], 10.1601/nm.2200 (formerly 10.1601/nm.1877) was recognized as a new species in 1988 [26]. The species epithet derives from the Latin (al.ber.ten’sis. M.L. adj. *albertensis* Albertan), meaning pertaining to Alberta, a province of Canada, from where it was first isolated. The type strain is 10.1601/strainfinder?urlappend=%3Fid%3DDSM+14366/10.1601/strainfinder?urlappend=%3Fid%3DATCC+35403. 10.1601/nm.2200 was described as a mesophilic, obligatory autotrophic sulfur-oxidizer that did not oxidize iron. Differentiating characteristics from other members of the acidithiobacilli include forming yellowish colonies on solid sulfur-containing media, a slightly larger cellular size, a tuft of polar flagella, a glycocalyx and a number of large intracellular sulfur globules [5, 17]. 10.1601/nm.2200 was reported to have a more confined pH range for growth (2–4.5) and a slightly higher temperature growth optimum with respect to other members of the genus [1], although these features may vary between strains [17]. Additional properties of 10.1601/nm.2200 are listed in Table 1.

**Table 1:** Classification and general features of *A. albertensis* strain^T^ [22]

MIGS ID	Property	Term	Evidence code^a^
	Classification	Domain *Bacteria*	TAS [1]
		Phylum *Proteobacteria*	TAS [1]
		Class *Acidithiobacillia*	TAS [2]
		Order *Acidithiobacillales*	TAS [47, 48]
		Family *Acidithiobacillaceae*	TAS [47, 49]
		Genus *Acidithiobacillus*	TAS [1]
		Species *Acidithiobacillus albertensis*	TAS [5, 26]
		(Type) strain: *Strain* ^*T*^ *(DSM 14366)*	
	Gram stain	Negative	TAS [5]
	Cell shape	Rod	TAS [5]
	Motility	Motile	TAS [5]
	Sporulation	Not reported	NAS
	Temperature range	10–40 °C	TAS [5]
	Optimum temperature	25–30 °C	TAS [5]
	pH range; Optimum	2.0–4.5; 3.5–4.0	TAS [5]
	Carbon source	CO_2_	TAS [5]
MIGS-6	Habitat	Acidic mineral-sulfur rich environments	TAS [5]
MIGS-6.3	Salinity	Not reported	NAS
MIGS-22	Oxygen requirement	Aerobic	TAS [5]
MIGS-15	Biotic relationship	Free-living	NAS
MIGS-14	Pathogenicity	Non-pathogen	NAS
MIGS-4	Geographic location	Canada/Alberta	TAS [5]
MIGS-5	Sample collection	1983	TAS [5]
MIGS-4.1	Latitude	Not reported	NAS
MIGS-4.2	Longitude	Not reported	NAS
MIGS-4.4	Altitude	Not reported	NAS

^a^Evidence codes – *IDA* Inferred from Direct Assay, *TAS*: Traceable Author Statement (i.e., a direct report exists in the literature), *NAS* Non-traceable Author Statement (i.e., not directly observed for the living, isolated sample, but based on a generally accepted property for the species, or anecdotal evidence). These evidence codes are from the Gene Ontology project [50]

**Fig. 1:**
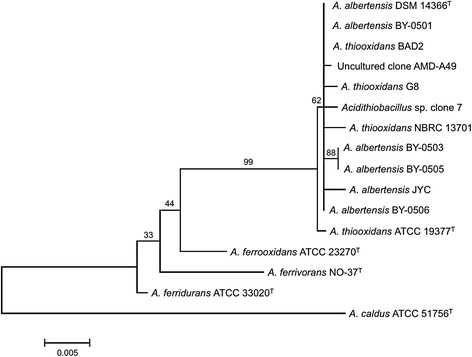
Phylogenetic tree based on 16S rDNA sequence information position of *A. albertensis* strain DSM 14366^T^ (type strain = ^T^) relative to other type and non-type strains within the acidithiobacilli. The strains and their corresponding GenBank accession numbers for 16S rRNA genes are: *A. albertensis* DSM 14366^T^, NR_028982; *A. albertensis* BY0501, FJ032185; *A. albertensis* BY0503, FJ032186; *A. albertensis* BY0505, FJ032187; *A. albertensis* BY0506, GQ254658; *A. albertensis* JYC, FJ172635; *A. thiooxidans* ATCC 19377^T^, Y11596; *A. thiooxidans* BAD2, KC902821; *A. thiooxidans* G8, KC902819; *A. thiooxidans* NBRC13701, AY830902, AMD uncultured clone c7, JX989232; *A. ferrooxidans* ATCC 23270^T^, NR_074193; *A. ferrivorans* NO-37, NR_114620; *A. ferridurans* ATCC 33020 ^T^, NR_117036; *A. caldus* ATCC 51756 ^T^, CP005986. The tree was inferred using the Neighbor-Joining method [51]. The optimal tree with the sum of branch length = 0.08720008 is shown. The percentage of replicate trees in which the associated taxa clustered together in the bootstrap test (1000 replicates) are shown next to the branches. The tree is drawn to scale, with branch lengths in the same units as those of the evolutionary distances used to infer the phylogenetic tree. The evolutionary distances were computed using the Maximum Composite Likelihood method [52] and are in the units of the number of base substitutions per site. The analysis involved 34nucleotide sequences. There were a total of 1314 positions in the final dataset. Evolutionary analyses were conducted in MEGA6 [53]

Phylogenetic analysis of the 16S rRNA gene sequence of 10.1601/nm.2200 10.1601/strainfinder?urlappend=%3Fid%3DDSM+14366 ^T^ places the type strain close to a few other cultivated members of the species and several uncultured clones deposited in GenBank, all of which are 100% identical at the16S rRNA gene level (Fig. 1). The 10.1601/nm.2200 type strain and its closest relatives branch apart from 10.1601/nm.2199 ^T^.

## Genome sequencing information

### Genome project history

The organism was selected for sequencing on the basis of its phylogenetic position and 16S rRNA similarity to members of the genus 10.1601/nm.2198. This represents the first draft genome sequence of an 10.1601/nm.2200 strain. The Whole Genome Shotgun project has been deposited at GenBank under the accession MOAD00000000. The version described in this paper consists of 1 scaffold (2.7 > X Mbp) and 140 smaller contigs and is the first version, MOAD01000000. Table 2 presents the project information and its association with MIGS (version 2.0) compliance [27].

**Table 2:** Project information

MIGS ID	Property	Term
MIGS 31	Finishing quality	Draft
MIGS-28	Libraries used	Nextera 2.1
MIGS 29	Sequencing platforms	Illumina MiSeq
MIGS 31.2	Fold coverage	64 x
MIGS 30	Assemblers	Velvet v 1.2.10
MIGS 32	Gene calling method	Glimmer 3.02
	Locus Tag	BLW97
	Genbank ID	MOAD00000000
	GenBank Date of Release	FEB 15, 2017
	GOLD ID	Gp0225628
	BIOPROJECT	PRJNA351776
MIGS 13	Source Material Identifier	DSM 14366
	Project relevance	Biomining, Tree of life

### Growth conditions and genomic DNA preparation

10.1601/nm.2200 strain 10.1601/strainfinder?urlappend=%3Fid%3DDSM+14366 ^T^ was obtained from the DSMZ collection and grown in 10.1601/strainfinder?urlappend=%3Fid%3DDSMZ+71 medium at 30 °C. DNA isolation and routine manipulations were carried out following standard protocols [28].

### Genome sequencing and assembly

The genome of 10.1601/nm.2200 10.1601/strainfinder?urlappend=%3Fid%3DDSM+14366 ^T^ was sequenced using Illumina sequencing technology (MiSeq platform) and paired-end libraries. Duplicate high quality libraries with insert sizes of ~460 bp were prepared using Nextera™ DNA Sample Preparation kit (Nextera, USA). Raw sequencing reads were preprocessed using Trimmomatic v0.32 [29]. Only reads with a quality score > Q30 (corresponding to less than 1 error per 1000 bp) and a read length > 35 nt were retained. High quality reads were assembled de novo using Velvet (v1.2.10) [30] and a k-mer length of 151, with an N50 of 39,225. Contig segments with at least 37 fold coverage were further scaffolded. The final draft assembly contained 1 scaffold (2.7 > X Mbp) and 140 smaller contigs. The total size of the draft genome is ~3.1 Mbp and the final assembly is based on 3.1 Gbp of Illumina data.

### Genome annotations

Genes were identified using Glimmer 3.02 [31] as part of the RAST annotation pipeline [32]. The tRNA and tmRNA predictions were made using ARAGORN v1.2.36 [33] and the rRNA prediction was carried out via HMMER3 [34]. Additional gene prediction analysis and manual functional annotation curation was performed using in house resources. The predicted CDSs were used to search the National Center for Biotechnology Information non-redundant database, UniProt, TIGRFam, Pfam, PRIAM, KEGG, COG and InterPro databases. Protein coding genes were analyzed for signalpeptides using SignalP v4.1 [35] and transmembrane helices using TMHMM v2.0 [36]. The circular map was drawn with CGView [37]. Single nucleotide polymorphisms were called using SNAP v2.1.1 [www.hiv.lanl.gov/content/sequence/SNAP/SNAP.html]. Non-synonymous substitution rates were calculated as the proportion between the number of observed synonymous substitutions in pairwise gene alignments and the size of the each alignment, and are expressed in percent.

Genome comparisons were performed using the GET_HOMOLOGUES software package (version 07112016). Orthology was determined based on all-versus-all Best Bidirectional BlastP Hit and COGtriangles v2.1 as clustering algorithm. Pairwise alignment cutoffs were set at 75% coverage and *E*-value of 10E-5. The phylogenomic relationships between the 10.1601/nm.2200 ^T^ and other 10.1601/nm.2198 strains were inferred from the average nucleotide identity (ANI) values assessed by BLASTn [38] and the in silico DNA-DNA hybridization indexes (DDH) assessed using the Genome-to-Genome Distance Calculator with recommended formula 2 [39]. Species cutoff limits were those defined by Meier-Kolthoff and colleagues [40].

## Genome properties

**Fig. 2:**
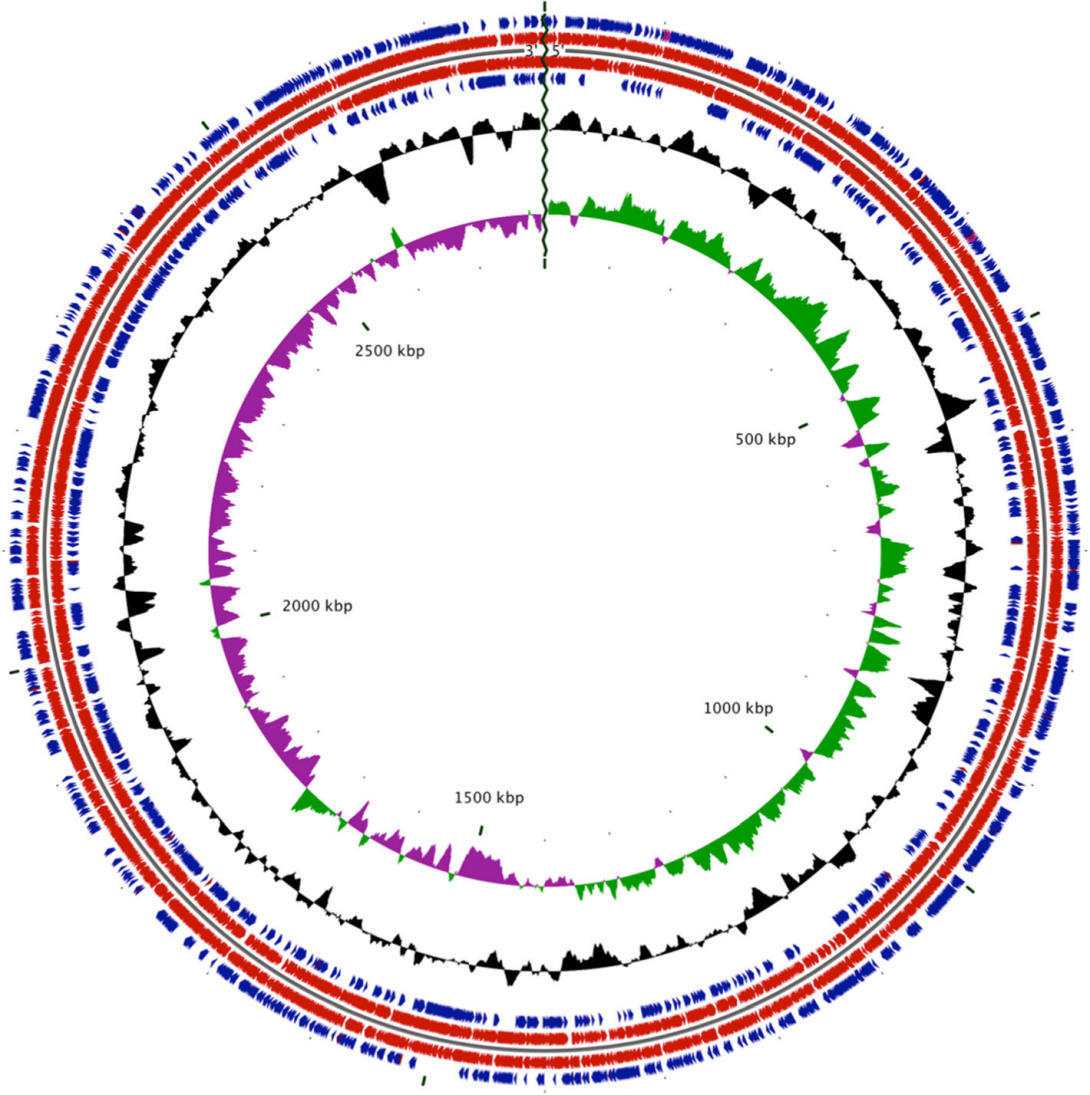
Circular representation of the high quality draft genome of *A. albertensis* ^T^ displaying relevant genome features. The features are the following (from outside to inside): Genes on forward strand (red); Genes on reverse strand (red); CDSs (blue), GC content (black); GC skew (green and purple)

The 3.5 Mbp draft genome of 10.1601/nm.2200 ^T^ is currently arranged into one high quality scaffold (Fig. 2) and 140 smaller contigs, most of which correspond to fragments of plasmids and other mobile genetic elements. According to the criteria of conservation of universal housekeeping genes [41], the genome is predicted to be 99.9% complete. Its average G + C content is 52.5% (Table 3). From a total of 3202 predicted genes, 3149 were protein-coding genes and 53 were RNA genes. A total of 63.4% of the CDSs were assigned a putative function while the remainders were annotated as hypotheticals. A total of 53 RNA genes partitioned into 47 tRNAs, 1 tmRNA and 2 rRNA operons (Table 3). The presence of two rRNA operons has recently been experimentally validated [25]. According to the genomic sequence information, the two operons are 100% identical. The distribution of genes into COGs functional categories is presented in Table 4.

**Table 3:** Genome statistics

Attribute	Value	% of Total^a^
Genome size (bp)	3,497,418	100.00
DNA coding (bp)	2,930,787	83.80
DNA G + C (bp)	1,836,144	52.50
DNA scaffolds	141	100.00
Total genes^b^	3202	100.00
Protein coding genes	3149	98.34
RNA genes^c^	53	1.66
Pseudo genes	n.d	n.d
Genes in internal clusters	n.d	n.d
Genes with function prediction	1967	61.43
Genes assigned to COGs	2322	72.52
Genes with Pfam domains	2152	67,21
Genes with signalpeptides	374	11.68
Genes with transmembrane helices	727	22.70
CRISPR repeats	0	0

^a^The total is based on either the size of the genome in base pairs or the total number of genes in theannotated genome

^b^Includes tRNA, tmRNA, rRNA

^c^Includes 23S, 16S and 5S rRNA

**Table 4:** Number of genes associated with general COG functional categories

Code	Value	%age	Description
J	135	4.22	Translation
A	1	0.03	RNA processing and modification
K	124	3.87	Transcription
L	181	5.65	Replication, recombination and repair
B	1	0.03	Chromatin structure and dynamics
D	29	0.91	Cell cycle control, mitosis and meiosis
Y	0	0.00	Nuclear structure
V	52	1.62	Defense mechanisms
T	127	3.97	Signal transduction mechanisms
M	203	6.34	Cell wall/membrane biogenesis
N	66	2.06	Cell motility
Z	0	0.00	Cytoskeleton
W	0	0.00	Extracellular structures
U	102	3.19	Intracellular trafficking and secretion
O	104	3.25	Posttranslational modification, protein turnover, chaperones
C	169	5.28	Energy production and conversion
G	113	3.53	Carbohydrate transport and metabolism
E	156	4.87	Amino acid transport and metabolism
F	53	1.66	Nucleotide transport and metabolism
H	102	3.19	Coenzyme transport and metabolism
I	57	1.78	Lipid transport and metabolism
P	109	3.40	Inorganic ion transport and metabolism
Q	37	1.16	Secondary metabolites biosynthesis, transport and catabolism
R	222	6.93	General function prediction only
S	179	5.60	Function unknown
–	880	27,48	Not in COGs

The total is based on the total number of predicted protein coding genes in the annotated genome

## Insights from the genome sequence

Metabolic reconstruction analysis revealed a complete suite of genes for sulfur oxidation, including those encoding the SOX complex (*soxYZB-AX* and *soxYZA-B, soxH*), tetrathionate hydrolase (*tetH, doxD*) and heterodisulfide reductase (*hdrBC and hdrABC*) previously found in 10.1601/nm.2199 ^T^ and 10.1601/nm.2201 ^T^ [42, 43]. Multiple copies of cytochrome d (*cydAB*) and cytochrome o (*cyoACBD*) terminal oxidases found in professional sulfur-oxidizing acidithiobacilli [19], also occur in 10.1601/nm.2200 ^T^. Genes for carbon dioxide fixation are well conserved, but no genes for nitrogen fixation were detected in the draft genome. Instead, genes for nitrate/nitrite assimilation and urea hydrolysis, both resulting in the production of ammonia, were found in the genome of the 10.1601/nm.2200 ^T^, along with a number of ammonia transporters.

**Fig. 3:**
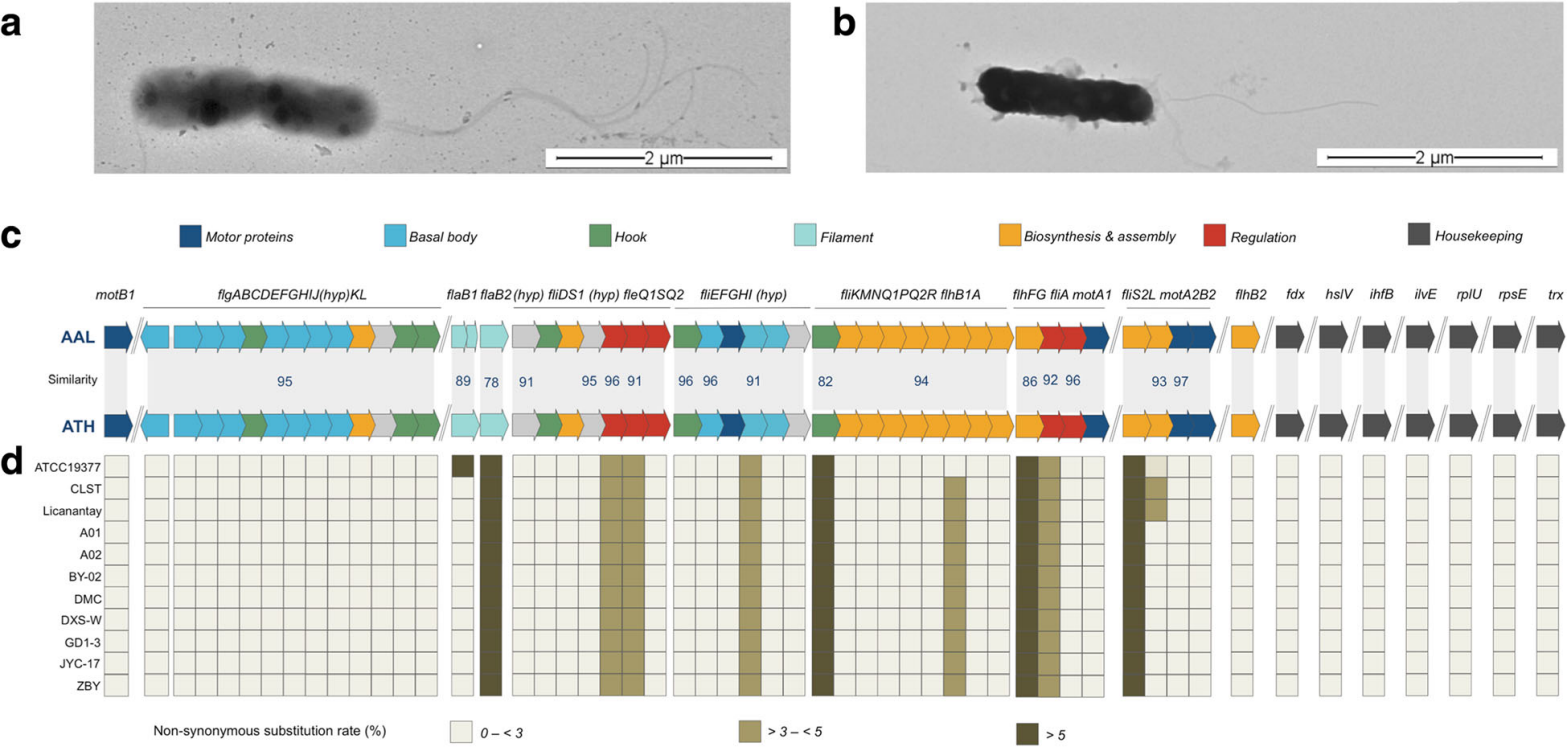
Flagellation patterns in *A. albertensis* ^T^ and *A. thioxidans* ^T^. **a** Transmission electron micrograph showing a dividing *A. albertensis* DSM 14366^T^ cell with tuft of polar flagella and **b** a cell of *A. thiooxidans* ATCC 19377^T^ with a single polar flagellum. **c** Comparison of the flagellar gene cluster between *A. albertensis* ^T^ (AAL) and *A. thiooxidans* ^T^ (ATH) derived from the corresponding genomic sequences. Flagellar genes and gene clusters are indicated accordingly. Percentage of amino-acid similarity is indicated only when bellow 98%. Color coding is as follows: motor proteins (blue), basal-body (turquoise), hook (green), flagellin (light blue), biosynthesis and assembly functions (orange), regulation (red). **d** Heatmap of the non-synonymous amino acidic substitution rates (percent) of the protein products of each flagellar gene and seven housekeeping genes from *A. albertensis* ^T^ and 11 *A. thioxidans* ^T^ sequenced strains (AFOH01, LGYM01, JMEB01, AZMO01, LWSA01, LWRZ01, LWSB01, LWRY01, LWSC01, LWSD01, LZYI01). Housekeeping genes were chosen after Nuñez et al. [25]

Gene clusters for the biosynthesis and assembly of flagella, which is a differential morphologic trait between this species and 10.1601/nm.2199, are conserved with respect to those encoded in the latter, in both general architecture and gene content. The pairwise identity between the predicted protein products of the flagellar genes of both type strains ranges from 87 to 100%, suggesting as well, the common ancestry of the operons. Yet, a relevant number of SNPs (single nucleotide polymorphisms) producing non-synonymous amino acidic substitutions of presently unclear relevance were uncovered in nine genes of the 10.1601/nm.2200 ^T^ flagellar cluster (Fig. 3), namely: *flaB2*, *flhF*, *flhG*, *fliH*, *fliK*, *fliR*, *fliS2*, *fleS* and *fleQ1*. All these genes are well conserved between 10.1601/nm.2199 strains (Fig. 3). The gene variants identified in 10.1601/nm.2200 were validated by read recruitment on a one-to-one basis, and are supported by more than 75 fold average (deep) coverage. These genes encode the flagellins FlaB2, the hook-length control protein FliK, the biosynthesis proteins FlhF, FliR and FliS, the biosynthesis regulator FlhG, also known as FleN, the assembly protein FliH, the sensor histidine kinase FleS and the regulator FleQ. Among these proteins, FlhF and FlhG/FleN encode proteins that have been shown to be relevant in the control flagellation patterns in other model bacteria [44], suggesting that differences in flagellation between 10.1601/nm.2200 (lophotrichous) and 10.1601/nm.2199 (monotrichous) shown in Fig. 3 might be partially attributed to divergence in these genes (6–14%). For the rest of the flagellar genes the rate of SNPs conductive to amino acidic substitutions between 10.1601/nm.2200 and other 10.1601/nm.2199 sequenced strains is low (<3) and similar to the rate observed in well conserved housekeeping genes. Further studies should be pursued to clarify the relevance of the uncovered substitutions in the flagellation patterns of the acidithiobacilli. Also, a larger number of chemotaxis genes were predicted in the 10.1601/nm.2200 ^T^ genome sequence with respect to those in 10.1601/nm.2199. This latter set of genes is organized in a cluster that includes *mcp1-cheYSA-mcp2-cheWRDB*, and encodes proteins participating in sensory adaptation to changing environmental signals rather than flagellar motor control [45].

Differences between the 10.1601/nm.2200 ^*T*^ genome and the pangenome of 10 other sequenced 10.1601/nm.2199 (recently reported by [22]) can be attributed to little over 1000 genes (1066 genes). Nearly half of these genes pertain to at least 10 integrated mobile genetic elements and a presently unclear number of plasmids, representing up to 16.2% of the 10.1601/nm.2200 ^*T*^ genome. In these genomic segments 54.5% of the genes are hypotheticals but a number of relevant functions were also detected, including among others: a) four orthologs of the sulfur oxygenase reductases (*sor1–4*), b) the gene cluster encoding the assimilatory nitrate and nitrite reductases, c) the urea carboxylase/allophanate hydrolase and the urea ABC transporter encoding genes, d) the spermidine/putrescine ABC transporter *potABC* and e) the three-gene operon associated with rubrerythrin, recently described by Cárdenas et al. [46]. All of these functions could confer adaptive advantages to 10.1601/nm.2200 ^T^ over 10.1601/nm.2199 strains under nitrogen and oxygen limitation and/or under extremely low pH.

Differences in gene dosage have also been observed between the two mesophilic sulfur-oxidizing/non iron-oxidizing species based on the comparison of the two type strains. 10.1601/nm.2200 ^T^ has more copies or gene variants (2 to more than 30) of the following: a) transposases and inactivated derivatives, b) thiol:disulfide interchange protein DsbG precursor, c) methyl-accepting chemotaxis receptor proteins, d) Crp/Fnr, LysR and MerR family transcriptional regulators, e) cytochrome d ubiquinol oxidases and e) SOR sulfur oxygenase reductases. The latter occur in four copies in the 10.1601/nm.2200 ^T^ genome, being completely absent in 10.1601/nm.2199 ^T^. Also more than 30 predicted protein products with GGDEF/EAL domains, likely involved in nucleotide driven signaling pathways, control and modulate gene expression and/or activity in 10.1601/nm.2200 ^T^, 40% of which seem to be exclusive to this species. Significant quantitative and qualitative differences in gene content have been reported before between strains of 10.1601/nm.2199 obtained from industrial processes [21, 22].

Despite the above mentioned differences between the type strains of 10.1601/nm.2200 and 10.1601/nm.2199, the average nucleotide identity value assessed by BLASTn (97,4%) and the in silico DNA-DNA hybridization index assessed by GGDC (82.9%) are bellow the currently recognized species cutoff limits [39], implying that 10.1601/nm.2200 and 10.1601/nm.2199 probably comprise a single genospecies.

## Conclusions

Altogether, the evidence presented herein suggests that validity of 10.1601/nm.2200 as an independent species should be reconsidered. In this respect, genomic approaches are crucial for understanding evolutionary processes and the origins of microbial biodiversity. The availability of the first high quality draft genome sequence of an 10.1601/nm.2200 strain will certainly enable more comprehensive comparative genomic studies and contribute to the resolution of the taxonomy and phylogeny of the genus. From a genomic standpoint, further analyses should be performed to assess if existing differences between the two type strains extend to other strains of each ‘presumed species’.

## Abbreviations

ATCC: American Type Culture Collection
DSMZ: Deutsche Sammlung von Mikroorganismen und Zellkulturen
NCBI: National Center for Biotechnology Information
